# The mitochondrial genome of *Protostrongylus rufescens* – implications for population and systematic studies

**DOI:** 10.1186/1756-3305-6-263

**Published:** 2013-09-12

**Authors:** Abdul Jabbar, Namitha Mohandas, Aaron R Jex, Robin B Gasser

**Affiliations:** 1 Faculty of Veterinary Science, The University of Melbourne, Parkville, Melbourne, Vic 3010, Australia

**Keywords:** *Protostrongylus* (Nematoda: Strongylida), Lungworm, Protostrongylosis, Sheep, Mitochondrial genome, Systematics, Epidemiology

## Abstract

**Background:**

*Protostrongylus rufescens* is a metastrongyloid nematode of small ruminants, such as sheep and goats, causing protostrongylosis. In spite of its importance, the ecology and epidemiology of this parasite are not entirely understood. In addition, genetic data are scant for *P. rufescens* and related metastrongyloids.

**Methods:**

The mt genome was amplified from a single adult worm of *P. rufescens* (from sheep) by long-PCR, sequenced using 454-technology and annotated using bioinformatic tools. Amino acid sequences inferred from individual genes of the mt genomes were concatenated and subjected to phylogenetic analysis using Bayesian inference.

**Results:**

The circular mitochondrial genome was 13,619 bp in length and contained two ribosomal RNA, 12 protein-coding and 22 transfer RNA genes, consistent with nematodes of the order Strongylida for which mt genomes have been determined. Phylogenetic analysis of the concatenated amino acid sequence data for the 12 mt proteins showed that *P. rufescens* was closely related to *Aelurostrongylus abstrusus*, *Angiostrongylus vasorum*, *Angiostrongylus cantonensis* and *Angiostrongylus costaricensis*.

**Conclusions:**

The mt genome determined herein provides a source of markers for future investigations of *P. rufescens*. Molecular tools, employing such mt markers, are likely to find applicability in studies of the population biology of this parasite and the systematics of lungworms.

## Background

*Protostrongylus rufescens* is a metastrongyloid nematode of small ruminants, including sheep and goats (definitive hosts) in most parts of the world [1]. The dioecious adults of this nematode live in the respiratory system (terminal bronchioles and alveoli) of the definitive host. Here, the females produce eggs, from which first-stage larvae (L1s) hatch within the airways of the lung. L1s then migrate *via* the bronchial/tracheal escalator to the pharynx, are swallowed and are then excreted in the faeces. L1s infect a molluscan intermediate host (snail) and then develop, under favourable environmental conditions, into third-stage larvae (L3) [1]. L3s within an infected intermediate host are then ingested by the ruminant host, penetrate the gut wall and then migrate *via* the lymphatic system or blood stream to the lungs, where they develop to adult worms. The prepatent period is reported to be ~ 4–9 weeks [2]. Although *P. rufescens* infection is widespread, it does not usually cause major clinical disease. Nonetheless, pathological changes, characterized by chronic, eosinophilic, granulomatous pneumonia, can be detected upon *post mortem* examination. Adult worms reside mainly in the bronchioles and alveoli, and are surrounded by macrophages, giant cells, eosinophils and other inflammatory cells which produce grey or beige plaques (1–2 cm) under the pleura in the dorsal border of the caudal lung lobes [3].

Little is known about fundamental aspects of the epidemiology and ecology of *P. rufescens*. Molecular tools employing suitable genetic markers can underpin fundamental studies in these areas, with a perspective on investigating transmission patterns linked to particular genotypes of a parasite and on discovering population variants or cryptic species [4,5]. Advances in nucleic acid sequencing and bioinformatics have provided a foundation for characterizing the mt genomes from parasitic nematodes as a source of genetic markers for such explorations. Here, we used an established, massively parallel sequencing-bioinformatics pipeline [6] for the characterization of the mt genome of *P. rufescens*, which we compared with those of related metastrongyloid nematodes, for which mt genomic sequence data are available. We also studied the genetic relationships among these lungworms and selected representatives within the order Strongylida, and suggest that selected regions in the genome of *P. rufescens* should serve well as markers for future studies of the ecology and epidemiology of this nematode around the world.

## Methods

### Parasite and genomic DNA isolation

Adult worms of *P. rufescens* were collected from the lungs of a fresh sheep cadaver in Victoria, Australia, washed extensively in physiological saline and then stored at −80°C. Upon thawing, genomic DNA was isolated from a single adult male specimen using an established method of sodium dodecyl-sulphate (SDS)/proteinase K digestion and subsequent mini-column purification [7]. The identity of the specimen was verified by PCR-based sequencing (BigDye chemistry v.3.1) of the second internal transcribed spacer (ITS-2) of nuclear ribosomal DNA [7].

### Long-PCR, sequencing and mt genome assembly

From the genomic DNA extracted from the single male worm, the complete mt genome was amplified by long-PCR (BD Advantage 2, BD Biosciences) as two overlapping amplicons (~5 kb and ~10 kb), using the protocol described by Hu et al. [8], with appropriate positive (i.e., *Haemonchus contortus* DNA) and negative (i.e., no template) controls. Amplicons were consistently produced from the positive control samples; in no case was a product detected for the negative controls. Amplicons were then treated with shrimp alkaline phosphatase and exonuclease I [9], and quantified by spectrophotometry. Following agarose electrophoretic analysis, the two amplicons (2.5 μg of each) were pooled and subsequently sequenced using the 454 Genome Sequencer FLX (Roche) [10] according to an established protocol [6]. The mt genome sequence was assembled using the program CAP3 [11] from individual reads (of ~300 bp).

### Annotation and analyses of sequence data

Following assembly, the mt genome of *P. rufescens* was annotated using the bioinformatic annotation pipeline developed by Jex et al. [6]. Briefly, the open reading frame (ORF) of each protein-coding mt gene was identified (six reading frames) by comparison to those of the mt genome of *Angiostrongylus vasorum* [GenBank: JX268542; [12]]. The large and small subunits of the mt ribosomal RNA genes (*rrn*S and *rrn*L, respectively) were identified by local alignment. The transfer RNA (tRNA) genes were predicted (from both strands) based on their structure, using scalable models based on the standard mt tRNAs for nematodes [5]. Predicted tRNA genes were then grouped according to their anti-codon sequence and identified based on the amino acid encoded by the anti-codon. Two separate tRNA gene groups were predicted each for leucine (Leu) (one each for the anticodons CUN and UUR, respectively) and for serine (Ser) (one each for the anticodons AGN and UCN, respectively), as these tRNA genes are duplicated in many invertebrate mt genomes, including those of nematodes [5]. All predicted tRNAs for each amino acid group were ranked according to the “strength” of their structure (inferred based on minimum nucleotide mismatches in each stem); for each group, the 100 best-scoring structures were compared by BLASTn against a database comprising all tRNA genes for each amino acid for all published mt genome sequences of nematodes (available *via*http://drake.physics.mcmaster.ca/ogre/; [13]). The tRNA genes were then identified and annotated based on their highest sequence identity to known nematode tRNAs. Annotated sequence data were imported using the program SEQUIN (*via*http://www.ncbi.nlm.nih.gov/Sequin/), the mt genome structure verified and the final sequence submitted as an SQN file to the GenBank database.

### Phylogenetic analysis of concatenated amino acid sequence datasets

The amino acid sequences were predicted from individual mt genes of *P. rufescens* and of other nematodes, including *An. cantonensis*, *An. costaricensis*, *An. vasorum*, *Metastrongylus pudendotectus* and *M. salmi* [GenBank: GQ398122, JX268542, GQ398121, GQ888714 and GQ888715, respectively; Metastrongyloidea]; *Ancylostoma caninum* and *Necator americanus* [GenBank: FJ483518 and NC_003416, respectively; Anyclostomatoidea]; *H. contortus* and *Trichostrongylus axei* [GenBank: NC_010383 and GQ888719, respectively; Trichostrongyloidea]; *Oesophagostomum dentatum* and *Strongylus vulgaris* [GenBank: GQ888716 and GQ888717, respectively; Strongyloidea]; and *Strongyloides stercoralis* [GenBank: AJ558163; Strongyloidoidea] [6,12,14-20] (Table 1). All amino acid sequences were aligned using the program MUSCLE [21] and then subjected to phylogenetic analysis. For this analysis, best-fit models of evolution were selected using ProtTest 3.0 [22] employing the Akaike information criterion (AIC) [23]. Bayesian inference analysis was conducted using MrBayes 3.1.2 [24], with a fixed mtREV amino acid substitution model [25], using four rate categories approximating a Γ distribution, four chains and 200,000 generations, sampling every 100th generation. The first 200 generations were removed from the analysis as burn-in.

**Table 1:** Details of the whole mitochondrial genome sequences used in this study as reference sequences

**Species**	**Host**	**Predilection site**	**Length of genome (bp)**	**Accession number**	**Reference**
*Angiostrongylus vasorum*	Dog	Pulmonary artery	13422	NC_018602	[12]
*Aelurostrongylus abstrusus*	Cat	Lung	13913	JX519458	[28]
*Angiostrongylus cantonensis*	Rat	Pulmonary artery	13497	GQ398121	[20]
*Angiostrongylus costaricensis*	Rodent	Mesenteric arteries	13585	GQ398122	[20]
*Protostrongylus rufescens*	Sheep	Lung	13619	KF481953	This study
*Metastrongylus salmi*	Pig	Lung	13778	NC_013815	[6]
*Metastrongylus pudendotectus*	Pig	Lung	13793	NC_013813	[6]
*Haemonchus contortus*	Sheep	Abomasum	14055	NC_010383	[18]
*Trichostrongylus axei*	Sheep	Abomasum	13653	NC_013824	[6]
*Oesophagostomum dentatum*	Pig	Large intestine	13869	NC_013817	[6]
*Strongylus vulgaris*	Horse	Large intestine	14301	NC_013818	[6]
*Necator americanus*	Human	Small intestine-	13605	NC_003416	[15]
*Ancylostoma caninum*	Dog	Small intestine	13717	NC_012309	[19]
*Strongyloides stercoralis*	Dog	Small intestine	13758	AJ558163	[16]

## Results and discussion

### Features of the mt genome

**Figure 1:**
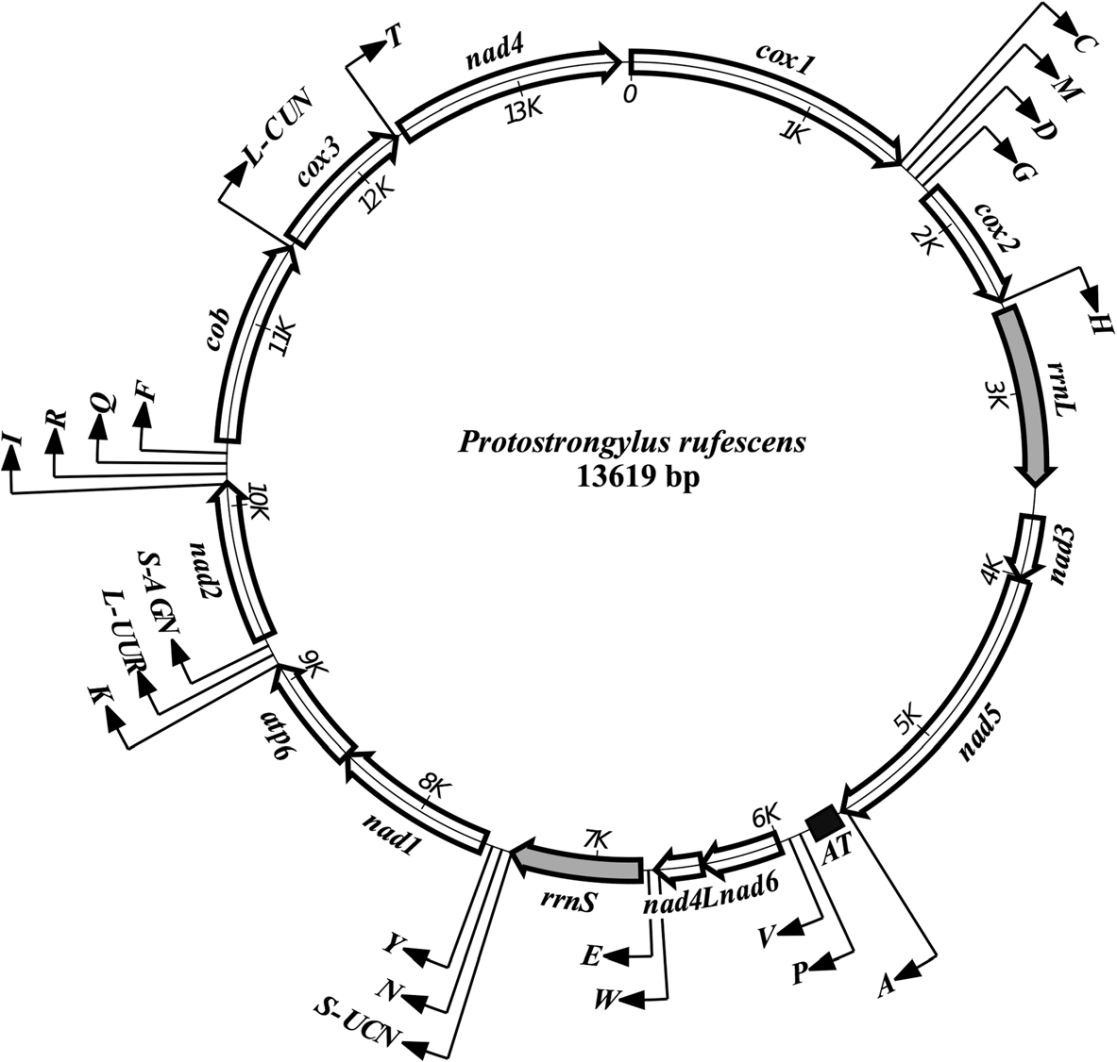
**Schematic representation of the circular mt genome of *Protostrongylus rufescens.*** Each transfer RNA gene is identified by a one-letter amino acid code in the map (external), and the AT rich region is also indicated. All genes are transcribed in the clockwise direction.

The circular mt genome sequence of *P. rufescens* [GenBank: KF481953] is 13,619 bp in length (Figure 1). It contains two ribosomal genes, 12 protein-coding (*cox*1-3, *nad*1-6, *nad*4L, *atp*6 and *cyt*b) and 22 tRNA genes. The gene arrangement (GA2) in the mt genome of *P. rufescens* was the same as all other strongylid nematodes studied to date [5,26]. All of the 36 genes are transcribed in the same direction (5′ to 3′) (Figure 1). Overall, the genome is AT-rich, as expected for strongylid nematodes [12,20,27,28], with T being the most favoured nucleotide and C the least favoured. The nucleotide contents were 25.9% (A), 6.8% (C), 18.6% (G) and 48.6% (T) (Table 2). The longest non-coding (AT-rich) region, located between the genes *trn*A and *trn*P, was 223 bp in length (see Figure 1); its AT-content was 83.4%, significantly greater than for all other parts of the mt genome (Table 2).

**Table 2:** **Nucleotide composition (%) for the entire or regions of the mitochondrial genome of *Protostrongylus rufescens***

**Nucleotide**	**Length (bp)**	**A**	**C**	**T**	**G**	**A + T**
Entire sequence	13619	25.9	6.8	48.6	18.6	74.5
Protein genes	10317	23.3	7.1	50.2	19.4	73.5
RNA genes	1661	33.1	6.3	44.2	16.4	77.3
AT-rich	223	44.8	5.8	10.7	38.6	83.4

### Ribosomal RNA genes

The *rrn*S and *rrn*L genes of *P. rufescens* were identified by sequence comparison with *An. vasorum*. The *rrn*S gene was located between *trn*E and *trn*S (UCN), and *rrn*L was between *trn*H and *nad*3. The two genes were separated from one another by the protein-encoding genes *nad*3, *nad*5, *nad*6 and *nad*4L (Figure 1). The sizes of the *rrn*S and *rrn*L genes of *P. rufescens* were 683 bp and 959 bp, respectively. The lengths of these two genes were similar to those of other metastrongyloids for which mt genomes are known (694–699 bp for *rrn*S, and 958–961 bp for *rrn*L [12,20,26-28] (Figure 1), and amongst the shortest for metazoan organisms [29].

### Protein-coding genes and codon usage

The prediction of initiation and termination codons for the protein-coding genes of *P. rufescens* (Table 3) revealed that the commonest start codon was ATT (for five of 12 proteins), followed by TTG (four genes), ATA (two genes) and ATG (one gene). Ten mt protein genes of *P. rufescens* were predicted to have a TAA or TAG translation termination codon. The other two protein genes ended in an abbreviated stop codon, such as T or TA (Table 3).

**Table 3:** **Summary of the mitochondrial genome of *Protostrongylus rufescens***

**Gene**	**Nucleotide positions**	**Sequence lengths**		**Codons**
		**No. of nucleotides**	**No. of amino acids encoded**	**Start/Stop**
*cox*1	1 – 1572	1571	523	ATA/TAA
*trn*C	1572 – 1626	54		
*trn*M	1626 – 1689	63		
*trn*D	1689 – 1742	53		
*trn*G	1744 – 1800	56		
*cox*2	1801 – 2493	692	230	ATT/TAA
*trn*H	2499 – 2553	54		
*rrn*L	2549 – 3508	959		
*nad*3	3682 – 4017	335	111	TTG/TAG
*nad*5	4022 – 5596	1574	524	ATT/T
*trn*A	5601 – 5655	54		
*trn*P	5878 – 5930	52		
*trn*V	5941 – 5994	53		
*nad*6	6004 – 6429	425	141	TTG/TAA
*nad*4L	6431 – 6664	233	77	ATT/T
*trn*W	6663 – 6718	55		
*trn*E	6719 – 6775	56		
*rrn*S	6776 – 7459	683		
*trn*S (UCN)	7461 – 7516	55		
*trn*N	7516 – 7569	53		
*trn*Y	7570 – 7623	53		
*nad*1	7621 – 8496	875	291	TTG/TAA
*atp*6	8497 – 9096	599	199	ATT/TAA
*trn*K	9104 – 9164	60		
*trn*L (UUR)	9165 – 9219	54		
*trn*S (AGN)	9220 – 9272	52		
*nad*2	9272 – 10129	857	285	ATT/TAG
*trn*I	10120 – 10177	57		
*trn*R	10175 – 10228	53		
*trn*Q	10231 – 10285	54		
*trn*F	10286 – 10342	56		
*cob*	10352 – 11455	1103	367	ATA/TAA
*trn*L (CUN)	11455 – 11510	55		
*cox*3	11499 – 12278	779	259	ATG/TAA
*trn*T	12274 – 12326	52		
*nad*4	12327 – 13556	1229	409	TTG/TAA

The codon usage for the 12 protein-encoding genes of *P. rufescens* was also compared with that of other metastrongyloid nematodes, *Aelurostrongylus* (*Ae.*) *abstrusus*, *An. cantonensis*, *An. costaricensis* and *An. vasorum*[12,20,28] (Table 4). All 64 codons were used. The preferred nucleotide usage at the third codon position of mt protein genes of *P. rufescens* reflects the overall nucleotide composition of the mt genome. At this position, T was the most frequently, and C the least frequently used. For *P. rufescens*, the codons ending in A had higher frequencies than the codons ending in G, which is similar to, for example, other members of the order Strongylida and *Caenorhabditis elegans* (Rhabditida), but distinct from *Ascaris suum* (Ascaridida) and *Onchocerca volvulus* (Spirurida) [14-17,30]. As the usage of synonymous codons is proposed to be preferred in gene regions of functional importance, codon bias appears to be linked to selection at silent sites and to translation efficiency [31,32].

**Table 4:** **Codon usages (%) in mitochondrial protein-encoding genes of *Protostrongylus rufescens***

***Amino acid***	***Codon***	***Number of codons and percentage (%) of codon usage***				
		***Protostrongylus rufescens***	***Angiostrongylus vasorum***	***Angiostrongylus cantonensis***	***Angiostrongylus costaricensis***	***Aelurostrongylus abstrusus***
**Non-polar**						
Alanine	GCN	98 (2.9)	88 (2.5)	75 (1.7)	52 (1.2)	84 (2.5)
Isoleucine	ATY	217 (6.3)	226 (6.5)	290 (6.4)	306 (6.8)	242 (7.1)
Leucine	CTN	47 (1.4)	23 (0.7)	135 (3.0)	152 (3.4)	66 (1.9)
Leucine	TTR	524 (15.5)	566 (16.4)	511 (11.3)	453 (10.1)	514 (14.9)
Methionine	ATR	202 (5.9)	148 (4.3)	225 (5.0)	191 (4.2)	103 (3.0)
Phenylalanine	TTY	495 (14.4)	461 (13.3)	614 (13.6)	675 (15.0)	548 (15.9)
Proline	CCN	71 (2.1)	71 (2.0)	57 (1.3)	35 (0.8)	75 (2.2)
Tryptophan	TGR	68 (1.9)	58 (1.7)	181 (4.0)	216 (4.8)	42 (1.2)
Valine	GTN	307 (8.9)	368 (10.6)	370 (8.2)	409 (9.1)	345 (10.1)
**Polar**						
Aspargine	AAY	118 (3.4)	92 (2.7)	146 (3.2)	155 (3.4)	98 (2.8)
Cysteine	TGY	45 (1.3)	77 (2.2)	156 (3.4)	209 (4.6)	59 (1.7)
Glutamine	CAR	39 (1.2)	38 (1.1)	46 (1.0)	32 (0.7)	43 (1.3)
Glycine	GGN	196 (5.7)	224 (6.5)	246 (5.4)	237 (5.3)	222 (6.5)
Serine	AGN	222 (6.5)	245 (7.1)	238 (5.3)	297 (6.6)	209 (6.1)
Serine	TCN	160 (4.7)	136 (3.9)	111 (2.5)	111 (2.4)	153 (4.5)
Threonine	ACN	96 (2.8)	77 (2.2)	102 (2.2)	56 (1.2)	80 (2.3)
Tyrosine	TAY	189 (5.5)	192 (5.5)	288 (6.4)	241 (5.4)	188 (5.5)
**Acidic**						
Aspartate	GAY	69 (2.0)	70 (2.0)	122 (2.7)	116 (2.6)	75 (2.2)
Glutamate	GAR	72 (2.1)	80 (2.3)	105 (2.3)	131 (2.9)	72 (2.1)
**Basic**						
Arginine	CGN	32 (0.9)	161 (4.6)	33 (0.7)	34 (0.7)	100 (2.9)
Histidine	CAY	52 (1.5)	53 (1.5)	48 (1.1)	36 (0.8)	55 (1.6)
Lysine	AAR	104 (3.3)	93 (2.7)	161 (3.6)	155 (3.4)	93 (2.7)

International Union of Pure and Applied Chemistry (IUPAC) codes (N = A, G, C or T; Y = C or T; R = A or G) were used.

The AT bias in the genome is also reflected in the amino acid composition of predicted proteins. The AT-rich codons represent the amino acids Phe, Ile, Met, Tyr, Asn the Lys, and GC-rich codons represent Pro, Ala, Arg the Gly. In the mt genome of *P. rufescens*, the most frequently used codons were TTT (Phe), TTA (Leu), ATT (Ile), TTG (Leu), TAT (Tyr), GGT (Gly), AAT (Asn) and GTT (Val). Six of these codons are AT-rich, and one of them is GC-rich. Seven of the eight codons contained an A or a T at two positions, except for GGT (Gly), which contained a T only in the third position. None of them had a C at any position. The least frequently used codons were CTC, CTG (Leu), GTC (Val), AGC (Ser), CCC (Pro), GCC (Ala), CAC (His), CGA (Arg), TCC (Ser), GGC (Gly) and ACC (Thr). All four GC-rich codons were represented here, and every codon had at least one C. When the frequencies of synonymous codons within the AT-rich group, such as Phe (TTT, 14.2%; TTC, 1.2%), Ile (ATT, 5.6%; ATC, 0.7%), Tyr (TAT, 5.6%; TAC, 0.9%) and Asn (AAT, 3.8%; AAC, 0.7%), were compared, the frequency was always less if the third position was a C.

### Transfer RNA genes

Twenty-two tRNA gene sequences were predicted in the mt genome of *P. rufescens*. These sequences ranged from 52–63 nt in length. The tRNA structures had a 7 bp amino-acyl arm, a 4 bp DHU arm, a 5 bp anticodon stem, a 7 base anticodon loop, a T always preceding an anticodon as well as a purine always following an anticodon. Twenty of the 22 tRNA genes (i.e. excluding the two *trn*S genes) have a predicted secondary structure with a 4 bp DHU stem and a DHU loop of 4–10 bases, in which the variable TψC arm and loop are replaced by a “TV-replacement loop” of 4–11 bases, in accordance with most nematodes whose mt genomes have been characterised [5]. The mt *trn*S for *P. rufescens* has a secondary structure consisting of a DHU replacement loop of 7 bases, 3 bp TψC arm, TψC loop of 4–6 bases and a variable loop of 3 bases, consistent with other members of the Chromadorea [6,14,20,33], but different from the enoplid nematodes *Trichinella murrelli* and *T. spiralis*[29,34,35]. Overlaps of one to four nucleotides are found between the genes *trn*H and *rrn*L*, nad*4L and *trn*W, *trn*Y and *nad*1, *trn*I and *trn*R within the mt genome of *P. rufescens*.

### Amino acid sequence comparisons and genetic relationships of *P. rufescens* with metastrongyloid and other nematodes

**Figure 2:**
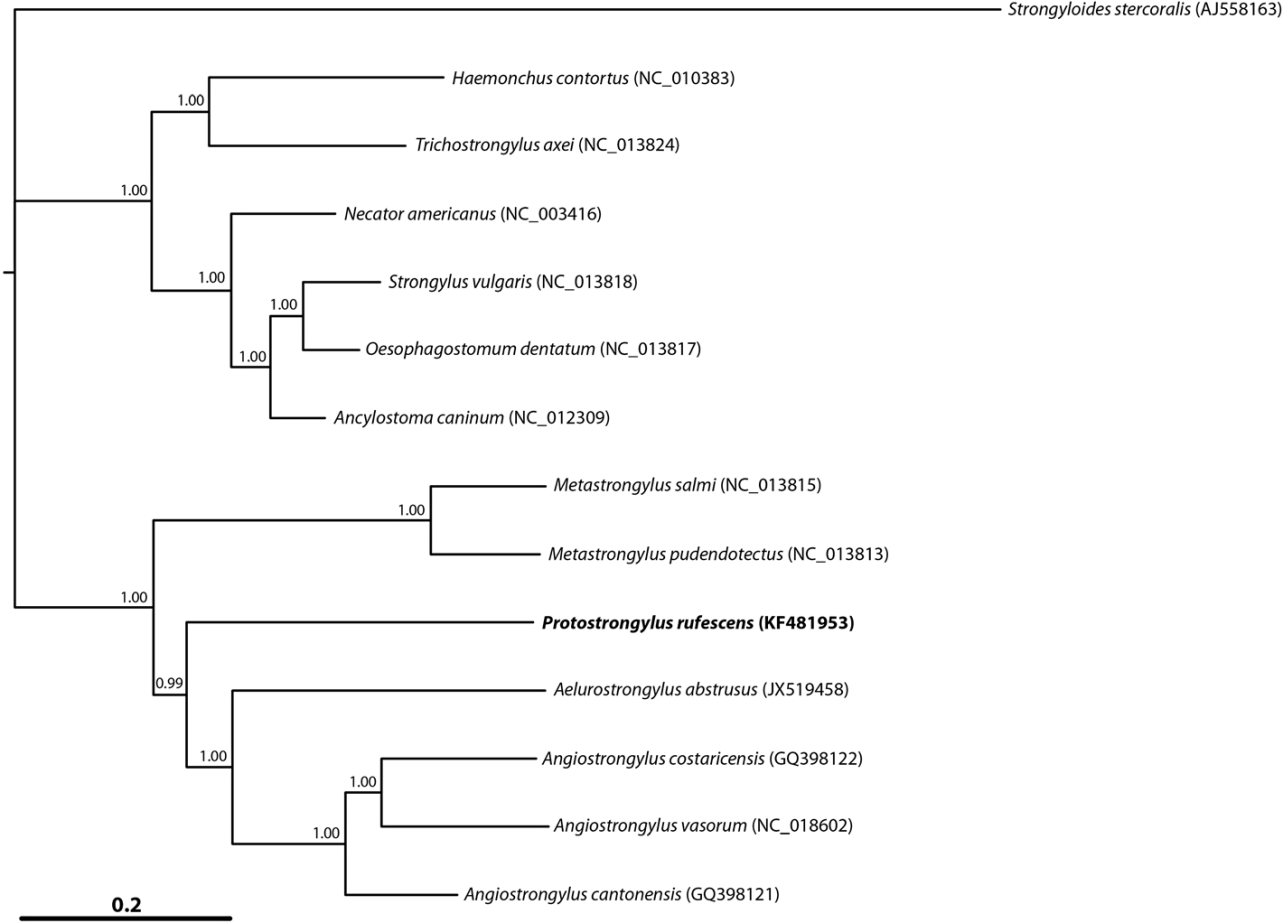
**Phylogenetic relationship of *Protostrongylus rufescens *with other nematodes.** Concatenated amino acid sequence data for all protein-encoding mitochondrial genes of *Protostrongylus rufescens* (bold) and other metastrongyloids, including *Aelurostrongylus abstrusus*, *Angiostrongylus cantonensis*, *An. costaricensis*, *An. vasorum*, *Metastrongylus pudendotectus* and *M. salmi* (metastrongyloids), as well as other concatenated sequence data representing different superfamilies, including *Ancylostoma caninum* and *Necator americanus* (hookworms; ancylostomatoids); *Haemonchus contortus* and *Trichostrongylus axei* (trichostrongyloids); *Oesophagostomum dentatum* and *Strongylus vulgaris* (strongyloids); and *Strongyloides stercoralis* (a rhabditid outgroup) were analyzed using Bayesian inference. The numbers above each tree branch represent the statistical support for each node (based on posterior probability [pp] score). GenBank accession numbers are in round brackets.

The amino acid sequences predicted from individual protein-encoding mt genes of *P. rufescens* were compared with those of *Ae. abstrusus*, *An. cantonensis*, *An. costaricensis, An. vasorum, Dictyocaulus viviparus* and *D. eckerti* (Table 5). Pairwise comparisons of the concatenated sequences revealed identities of 37.0-92.4% between these species. Based on identity, COX1 was the most conserved protein, whereas NAD2 and NAD6 were the least conserved. Phylogenetic analysis of the concatenated amino acid sequence data for the 12 mt proteins showed that *P. rufescens* was more closely related to *Ae. abstrusus*, *An. cantonensis*, *An. costaricensis* and *An. vasorum*, (pp = 1.00) than to *M. pudendotectus* and *M. salmi* (metastrongloids) (pp = 1.00), to the exclusion of *H. contortus, T. axei* (trichostrongyloids), *Anc. caninum*, *N. americanus* (hookworms; ancylostomatoids) and *O. dentatum* and *S. vulgaris* (strongyloids) (Figure 2) (pp = 1.00).

**Table 5:** Pairwise comparison of the amino acid sequences of the 12 protein-encoding mitochondrial genes

**Protein**	Amino acid identity (%)*					
	***Pr/Aa***	***Pr/Aca***	***Pr/Aco***	***Pr/Av***	***Pr/De***	***Pr/Dv***
ATP6	64.8	67.8	68.8	70.4	44.8	52.8
COB	71.7	70.4	70.7	71.7	65.7	65.1
COX1	92.4	91.0	90.0	89.1	86.4	86.2
COX2	80.0	77.4	75.2	72.2	63.5	66.7
COX3	82.2	82.0	81.5	73.8	69.9	70.0
NAD1	72.1	79.8	80.1	78.7	62.8	62.7
NAD2	55.0	57.0	58.8	53.0	37.0	43.0
NAD3	65.8	70.0	70.0	69.4	50.0	55.0
NAD4	68.9	68.7	68.5	66.3	55.6	52.9
NAD4L	76.9	74.0	68.8	64.0	51.8	64.1
NAD5	58.7	63.6	61.3	57.1	52.9	51.5
NAD6	58.0	58.2	59.4	56.0	46.0	38.0

*These percentages are based on comparisons (in %) of the amino acid sequences predicted from the mt genome of *Protostrongylus rufescens* (*Pr*) and those of related lungworms, *Aelurostronglyus abstrusus* (*Aa*)*, Angiostrongylus vasorum* (*Av*)*, Dictyocaulus eckerti* (*De*) and *Dictyocaulus viviparus* (*Dv*).

### Implications

The characterisation of the mt genome of *P. rufescens* provides genetic markers for future population genetic and systematic studies. As sequence variation in ITS-2 nuclear rDNA is usually low within most species of strongylid nematodes [36], mt DNA is better suited for assessing population genetic variation. Therefore, PCR-based analytical approaches, using *cox*1*, nad*1 and *nad*4 (displaying varying levels of within-species divergence), could be used to study haplotypic variation in *P. rufescens* populations in sheep and goats and also in molluscan hosts. Given that species complexes are commonly encountered in bursate nematodes [1,4,36], it would be interesting to prospect for cryptic species, to assess whether distinct genotypes/haplotypes of *P. rufescens* exist in sheep and goats as well as snails [37], and to establish whether particular sub-populations of *P. rufescens* occur in particular environments or geographical regions/countries, and have particular patterns of transmission.

It would also be interesting to assess the genetic structure of *P. rufescens* populations using PCR-coupled mutation scanning and sequencing of selected mt gene regions (such as *cox*1 and *nad*4), and mt DNA diversity within populations and the gene flow among populations. Findings for this lungworm (with an indirect life cycle *via* a molluscan intermediate host) could be compared with those for *D. viviparus* (with a direct life cycle), which has been reported to have surprisingly low mt DNA diversity within populations and limited gene flow among populations [38,39].

The complete mt genome of *P. rufescens* provides a basis for extended comparative mt genomic/proteomic analyses of other protostrongyloids of ruminants, including *P. brevispiculum*, *P. davtiani*, *P. hobmaieri*, *P. rushi*, *P. skrjabini*, *P. stilesi*, *Cystocaulus ocreatus*, *Neostrongylus lineatus*, *Muellerius capillaris* (the latter of which is a particularly pathogenic parasite in goats), and those of other animal hosts, such as lagomorphs and pinnipeds. Given the utility of predicted mt proteomic datasets, high phylogenetic signal and consistently high nodal support values in recent systematic analyses [6,12,27,28,33] provide an opportunity to reassess the evolutionary relationships of lungworms (order Strongylida). For example, the family Protostrongylidae is distinguished from other metastrongyloids by only a couple of morphological characters, i.e., the gubernaculm and telamon in adult male worms [40], and it is proposed that protostrongyloids of lagomorphs originated from their ancestors primarily infecting sheep, goat, antelopes and deer [41]. Analyses of inferred mt proteomic data sets from a range of protostrongyloids should allow relationships within the family Protostrongylidae and also the origin of the protostrongylids of lagomorphs to be assessed. In addition, there has been considerable debate as to the relationships among suborders within the Strongylida, based on the use of phenotypic characters [42]. On one hand, it has been hypothesized that the suborder Metastrongylina (to which species of *Protostrongylus*, *Metastrongylus*, *Aelurostrongylus* and *Angiostrongylus* belong) originated from ancestors in the Strongylina [43,44] or Trichostrongylina [45,46]. On the other hand, it has been proposed that the Metastrongylina gave rise to the Strongylina [47]. To date, molecular phylogenetic analyses of nuclear ribosomal rDNA sequence data [48,49] have suggested that the Trichostrongylina are basal to the Metastrongylina, which represented a monophyletic assemblage. However, Jex et al. [6], using mitochondrial sequence data, showed that the major suborders within the Strongylida (e.g., the Metastrongylina, Strongylina and Trichostrongylina) were each resolved as distinct, monophyletic clades with maximum statistical and nodal support (posterior probability = 1.00; bootstrap = 100). A detailed analysis using inferred mt proteomic data sets would allow an independent assessment of the systematic relationships of these suborders.

## Conclusions

Comparative analyses of proteomic sequence datasets inferred from the mt genomes of *P. rufescens* and other lungworms indicate that *P. rufescens* is closely related to *Ae. abstrusus*, *An. cantonensis*, *An. costaricensis* and *An. vasorum*. The mt genome determined herein should provide a source of markers for future investigations of *P. rufescens*. Molecular tools, employing such mt markers, are likely to find applicability in studies of the population biology of this parasite and the systematics of lungworms.

## Abbreviations

atp6: Adenosine triphosphatase subunit 6; cox: Cytochrome *c* subunit; cytb: Cytochrome *b* subunit; ITS-2: Second internal transcribed spacer; mt: Mitochondrial; nad: Nicotinamide dehydrogenase subunit; ORF: Open reading frame; rDNA: Nuclear ribosomal DNA; rrnL: Ribosomal large subunit; rrnS: Ribosomal small subunit; tRNA: Transfer RNA.

## Competing interests

The authors declare that they have no competing interests.

## Authors’ contributions

RBG & ARJ conceived the project and attracted the funding; AJ carried out molecular laboratory work; NM undertook the bioinformatics analysis; RBG, NM & AJ carried out data analysis and interpretation; RBG, AJ, NM & ARJ wrote the draft manuscript. All authors read and approved the final version of the manuscript.

## Authors’ information

Abdul Jabbar and Namitha Mohandas shared first authorship.
